# Effects of Selenium Sources on Meat Quality and Antioxidative Capacity in Donkeys: An Integrated Study Combining Cecal Microbiota Profiling and Multi-Omics Analysis

**DOI:** 10.3390/foods15203589

**Published:** 2026-10-09

**Authors:** Li Li, Yanli Zhao, Xiaoyu Guo, Yongmei Guo, Qingyue Zhang, Fanzhu Meng, Fang Hui, Sumei Yan

**Affiliations:** Key Laboratory of Animal Nutrition and Feed Science, Universities of Inner Mongolia Autonomous Region, Hohhot 010018, China; lily972021@163.com (L.L.); gxy_2594@163.com (X.G.); ymguo2015@163.com (Y.G.); alicezqy@126.com (Q.Z.); fzmeng2023@163.com (F.M.); cf18447051697@163.com (F.H.)

**Keywords:** meat donkeys, meat quality, antioxidant ability, selenium, sphingosine-1 phosphate

## Abstract

Our study investigated the improved nutritional quality and physicochemical properties in meat, as well as growth performance and antioxidant capacity in meat donkeys, from dietary selenium (Se) yeast (SY), modulating cecal microbiota and multi-omics profiles. A total of 16 one-year-old male meat donkeys (120 ± 25 kg) were allocated to two groups: SS (0.3 mg/kg inorganic Se from sodium Se) and SY (0.3 mg/kg organic Se from SY). SY supplementation significantly increased Se and crude protein content, water-holding capacity, and cooking loss, but reduced ether extract content, shear force, and drip loss of donkey meat; SY also improved body weight, nutrient digestibility, beneficial bacteria (*Agathobacter*, *Lactobacillus*), and antioxidant enzyme activities, while reducing pro-inflammatory cytokines and malondialdehyde. Metabolomics revealed upregulation of sphingosine-1 phosphate (S1P), and transcriptomics identified enrichment of the PI3K-Akt pathway. Validation confirmed that SY elevated S1P levels and upregulated S1P receptor 2 (S1PR2) and PI3K-Akt-related genes. In conclusion, meat quality and antioxidant capacity were enhanced, and cecal microbiota in donkeys were reshaped by dietary SY. These findings indicate that dietary selenium supplementation increases Se deposition and improves the quality of donkey meat, providing a promising approach for the development of Se-enriched, value-added functional donkey meat products and processed meat items.

## 1. Introduction

Consumers have become increasingly selective about what they eat, placing a premium on food safety and nutritional value over quantity. In this context, donkey meat has high crude protein, essential amino acids, and unsaturated fatty acids but low total fat and calories [1], which consumers highly favor. The nutrient content, such as protein, fat, and selenium (Se), as well as meat physicochemical properties, are the main indicators of meat quality. Specifically, water-holding capacity and cooking loss reflect the ability of muscle to retain water during processing and cooking; shear force is an indicator of tenderness; and color is a key appearance attribute influencing consumer acceptance, determining the nutritional value of meat. Therefore, intensive meat donkey farming has become inevitable to meet the huge market demand; consequently, this may lead to a decline in antioxidant levels. A decline in antioxidant status compromises performance parameters, including growth performance and meat quality [2]. What is more, dietary supplementation with natural antioxidants has been shown to elevate meat quality by enhancing antioxidant capacity [3]. Therefore, promoting antioxidant function and enhancing immune status are the basis for accelerated growth and meat quality in meat donkeys. Understanding how dietary SY modulates antioxidant pathways and cecal fermentation is therefore essential not only for animal performance but also for developing Se-fortified meat products that meet modern consumer demands for safer, more nutritious, and functionally enhanced foods.

Se is an indispensable trace mineral with multiple functions, including antioxidant, immune, and growth [4]. Se promotes meat quality, growth performance, and antioxidant function in cattle, sheep, pigs, and chickens [5,6,7,8], and commonly supplemented Se sources include Se yeast (SY), selenate, and selenite. Dietary SY is superior to inorganic Se in terms of tissue incorporation [9]. It is well established that SY can achieve a notable enhancement in Se content in milk, blood, and meat, and boost antioxidant defense [10,11,12,13]. Significant enhancement of meat quality—specifically lower cooking loss and shear force—is achieved through SY supplementation [14]. As a monogastric herbivore, the developed cecum of the donkey has a similar fermentation function as the rumen of ruminants. It is enriched with large numbers of microorganisms, which are the main site for digesting roughage. Therefore, investigating the antioxidant mechanisms of Se in donkeys, given their unique physiological structure, is particularly important. An Se-enriched diet can be conducive to rumen microflora establishment and enhance rumen microbial activity and digestive enzyme function [15]. However, the effects of dietary Se supplementation on meat donkeys and the differences in the impact of organic and inorganic Se have not been previously reported. Thus, we hypothesize that dietary organic Se enhances the donkey’s ability to counteract oxidative stress by: (1) promoting the colonization of beneficial cecal microbiota and thereby improving cecal fermentation, and (2) activating key antioxidant signaling pathways. This activation is proposed to potentiate the activity of antioxidant enzymes within the intestinal mucosa while concurrently reducing levels of malondialdehyde (MDA), reactive oxygen species (ROS), and pro-inflammatory cytokines. In parallel with enhanced antioxidant capacity and immune status, the meat quality of donkeys is also improved. Consequently, we intended to elucidate the regulatory response of dietary SY on growth and meat quality, and explore how this mechanism may be attributed to the elevation of cecal fermentation and antioxidant functions, as well as the alteration of cecal microorganisms and metabolites.

## 2. Materials and Methods

### 2.1. Donkeys, Diets, and Experimental Design

We used a single-factor completely randomized design. A total of 16 meat donkeys (male) aged 1 year old and similar in weight (120 ± 25 kg) were allocated to two treatment groups at random: SS group and SY group. The basal diet contained 0.019 mg/kg Se, the SS group added 0.3 mg/kg of inorganic Se to the basal diet (the source of Se was sodium Se), and the SY group added 0.3 mg/kg of organic Se (the source of Se was yeast Se, Na_2_SeO_3_) to the basal diet, respectively. The pre-test period lasted 10 days, followed by an 18-week trial period divided into three stages: early (weeks 1–6), middle (weeks 7–12), and late (weeks 13–18). The donkeys were fed 2 times a day and had free access to water at 8:00 a.m. and 3:00 p.m. After the test, all the donkeys were fasted for 12 h, remained water-free for 2 h, and were then slaughtered. Table 1 shows the basal diet composition and nutrient levels.

### 2.2. Growth Performance

All donkeys underwent morning weighing under fasting conditions prior to the experiment to determine initial body weight. Body weights were subsequently recorded after each fattening stage to compute the average daily gain (ADG) per period. Dry matter intake (DMI) was documented by replicate throughout the trial, and the feed-to-gain ratio (FGR) was then determined.

ADG = (End weight − start weight)/test days;

FGR = DMI/ADG.

### 2.3. Nutrient Digestibility

In the latter part of the fattening period, fecal samples were gathered three times a day (≥200 g per collection) over 7 consecutive days, and pooled per donkey. We also gathered feed samples, and both feed and fecal samples were assayed for crude protein (CP) and ether extract (EE) following AOAC procedures [16]. Neutral detergent fiber (NDF) and acid detergent fiber (ADF) were determined following Van Soest et al. [17]. Acid-insoluble ash (AIA) was employed to calculate the apparent digestibility, as described by Mehtiö et al. [18]:

Apparent digestibility (%) = 100 − [(FN × DA)/(DN × FA)] × 100.

where DN and FN represent the nutrient content (%) in the diet and feces, respectively, and DA and FA denote the AIA content (%) in the diet and feces, respectively.

### 2.4. Selenium Content, Physicochemical Properties, and Proximate Composition of Donkey Meat

Approximately 200 g of the left longissimus dorsi muscle was dissected from each donkey immediately after slaughter. Meat quality features, namely pH, color, water-holding capacity, cooking loss, shear force, and drip loss, were assessed based on the work of He et al. [19] and Zhang et al. [20]. Comprehensive methodological details are presented in the Supplementary Materials.

Se levels in the longissimus dorsi muscle were measured by a modified National Food Safety Standard method (GB5009.268-2016; Standards Press of China, Beijing, China) [21]. CP and EE content were determined following AOAC procedures [16].

### 2.5. Cecal Fermentation Variables

After the feeding experiment, all experimental donkeys were deprived of food for 12 h and denied water for 2 h before slaughter. Cecal contents were obtained, filtered, and the filtrate was retained for analysis. The pH value was immediately recorded with a portable pH meter. For NH3-N analysis, 0.5 mL filtrate was mixed with 4.5 mL of 0.2 mol/L HCl. For VFA analysis, 4 mL filtrate was mixed with 1 mL of 25% metaphosphoric acid, and the residual filtrate was retained for BCP analysis. VFAs (acetate, propionate, butyrate, isobutyrate, isovalerate, and valerate) were quantified by GC [22], and the A/P ratio was calculated. NH3-N was measured using the phenol hypochlorite method [23], and BCP was determined according to Makka’s method [22].

### 2.6. Serum Physiological Parameters in Serum, and Antioxidant and Immune Indicators in Duodenum and Cecum

Blood was collected and separated into serum for biochemistry measurements, including glucose (GLU), total cholesterol (CHO), triglyceride (TG), non-esterified fatty acids (NEFAs), high-density lipoprotein cholesterol (HDL-C), and low-density lipoprotein cholesterol (LDL-C), which were assayed by the standard kits (Nanjing Jiancheng Bioengineering Institute, Nanjing, China).

After slaughter, the duodenum and cecal mucosa were collected. Homogenates were prepared and then centrifuged to obtain supernatants for antioxidant and immune assays. The activities of catalase (CAT), GPx, total superoxide dismutase (T-SOD), and MDA concentration and total antioxidant capacity (T-AOC) in the duodenum and cecum were quantified by antioxidant kits (Nanjing Jiancheng Bioengineering Institute of China, Nanjing, China). The concentrations of interleukin (IL)-1β, IL-2, IL-6, IL-4, IL-10, tumor necrosis factor-alpha (TNF-α), nitric oxide (NO), inducible nitric oxide synthase (iNOS) and ROS, were assayed by the Beijing Sinouk Institute of Biological Technology, Beijing, China.

### 2.7. Cecal Microbiota, Metabolome, Transcriptome

Cecal contents were collected into vials (Corning, New York, NY, USA) for microbial diversity. Total microbial DNA was extracted using the E.Z.N.A.^®^ Soil DNA Kit (Omega Bio-Tek, Norcross, GA, USA) per the instructions. DNA quality and concentration were assessed by 1.0% agarose gel electrophoresis. The bacterial 16S rRNA V3–V4 region was targeted by primers 338F and 806R (with barcodes). Purified and quantified PCR products were run on an Illumina MiSeq PE300 platform (Illumina, San Diego, CA, USA) per standard protocols (Majorbio Bio-Pharm Technology Co., Ltd., Shanghai, China). Gut microbiota were analyzed on the Majorbio Cloud 2026 platform (https://cloud.majorbio.com) [24].

For metabolomics, we collected the cecal content into vials (Corning, New York, NY, USA). Sixteen cecum samples were analyzed on an LC-MS platform (Thermo Fisher Scientific UHPLC-Q Exactive HF-X system, Waltham, MA, USA, accessed on 21 October 2024), with QC samples injected every 8 runs for quality control. Raw LC/MS data were processed using Progenesis QI (Waters Corporation, Milford, MA, USA) and metabolites were identified against the HMDB, Metlin, and Majorbio databases. Data were then uploaded to the Majorbio cloud platform (https://cloud.majorbio.com, accessed on 21 October 2024) for further analysis.

For the transcriptome, we collected cecal mucosa into vials (Corning, New York, NY, USA, accessed on 21 October 2024). Briefly, total RNA was isolated from 50 mg of thawed cecal epithelial tissue samples using the TRIzol reagent (TaKaRa, Dalian, China) as described by the instructions. Only RNA samples meeting the following stringent quality criteria were used for subsequent library preparation: OD 260/280 of 1.8~2.2 and OD 260/230 ≥ 1.0, concentration ≥ 30 ng/μL, total quantity ≥ 1 μg, and RNA Integrity Number ≥ 6.5. For qualified RNA samples, transcriptome libraries were constructed using the Illumina NovaSeq Reagent Kit. Enrichment PCR was performed on the size-selected fragments, followed by final purification to obtain the cDNA libraries. Libraries were quantified using the TBS380 fluorometer (Picogreen assay). All sequencing services were processed by Majorbio Bio-Pharm Technology Co., Ltd. (Shanghai, China).

### 2.8. Integrated Analysis of the Cecal Metabolome and Transcriptome

Correlations were analyzed between the cecal transcriptome and metabolome data from this study. The co-enriched KEGG pathways identified through this integration were analyzed to identify pathways significantly enriched with both differentially expressed genes (DEGs) and differentially abundant metabolites (DAMs). These DEGs and DAMs were subsequently mapped onto their respective co-enriched KEGG pathways within the KEGG database.

### 2.9. S1P Content, S1P-Related Gene Expression, and PI3K-Akt Signaling Pathway Gene Expression in Cecal Mucosa

The content of sphingosine 1-phosphate (S1P) in the cecum mucosa was assayed employing commercial ELISA kits (Shanghai Enzyme-Linked Biotechnology Co., Ltd., Shanghai, China) as per the protocols. The relative expression levels of S1P receptor 2 (*S1PR2*), acid ceramidase (*ASAH1*), and sphingosine kinase 2 (*SPHK2*) in the cecum mucosa were measured by real-time quantitative PCR (qRT-PCR) analysis.

Cecal transcriptome analysis prompted validation of selected genes in the PI3K-Akt signaling pathway. And the mRNA expression of the guanine nucleotide-binding protein subunit gamma-T2 (*GNGT2*), phosphoinositide 3-kinase adapter protein 1 (*PIK3AP1*), phosphatidylinositol-4,5-bisphosphate 3-kinase catalytic subunit-alpha (*PIK3CA*), and RAC serine/threonine-protein kinase 1 (*Akt1*) was quantified by qRT-PCR analysis. The target gene mRNA relative quantity was calculated as 2^−∆∆Ct^ as per the comparative Ct method [25].

### 2.10. Statistical Analysis

We assessed growth performance using the PROC MIXED procedure of SAS (version 8.1, SAS Institute Inc., Cary, NC, USA). A mixed-effects model was applied, with the experimental group (SS, SY) and the fattening stage (FST) considered as fixed effects, and random effects included donkeys to account for repeated measurements. A compound symmetry (CS) covariance structure was used with donkeys as the subject, and the Kenward–Roger method was used to estimate denominator degrees of freedom. Student’s t-test served to evaluate nutrient digestibility, Se content, physicochemical properties, proximate composition of donkey meat, serum biochemical parameters, cecal fluid pH, BCP, NH_3_-N, VFA, antioxidant and immune indicators, alpha diversity indexes, phylum-level differential taxa, the content of S1P, and the mRNA expression of *S1PR2*, *ASAH1*, *SPHK2*, *GNGT2*, *PIK3AP1*, *PIK3CA* and *Akt1*. Significance was set at *p* < 0.05, and all data are presented as the mean ± SD, with 8 donkeys per group. LEfSe identified differentially abundant microbial genera (LDA score threshold = 2.9). Spearman correlation (R, heatmap package) was used to examine the relationship between growth performance and LEfSe-identified bacterial genera. Statistical significance was defined as *p* < 0.05.

## 3. Results

### 3.1. Growth Performance, Se Content, Physicochemical Properties, and Proximate Composition of Donkey Meat

Table 2 presents the growth performance data. The SY group displayed higher BW and ADG than the SS group (*p* < 0.001, *p* = 0.002). Se content, water-holding capacity, and cooking loss of the longissimus dorsi muscle were higher in SY versus the SS (*p* < 0.001, *p* < 0.001, *p* = 0.002), while the EE content, L*, b*, shear force, and drip loss displayed contrasting patterns (Table 3).

### 3.2. Serum Biochemical Parameters, Nutrient Digestibility, and Cecal Fermentation Function

As detailed in Table 4, the CHO concentration, the digestibility of CP, EE, and NDF, and that of SY were substantially greater in SY versus SS (*p* < 0.05), but LDL-C and NEFA concentrations were the opposite (*p* < 0.001). Relative to the SS group, the concentration of BCP, NH_3_-N, acetate, propionate, butyrate, isovalerate, valerate, and TVFAs was greater in the SY group (*p* < 0.05), but lower A/P. No significant differences were observed in ADF digestibility, TG and HDL-C content, pH, or isobutyrate content (*p* > 0.05).

### 3.3. Antioxidant and Immune Indicators in Duodenum and Cecum

We also determined antioxidant enzyme activities and concentrations of interleukin, MDA, NO, and ROS in the duodenum and cecal mucosa (Table 5). As presented in Table 4, relative to the SS group, SY had a higher level of CAT, GPx, T-SOD, T-AOC, and IL-2 in duodenum, and cecal mucosa, while reducing the iNOS activity and content of MDA, NO, IL-1β, IL-6, IL-10, and ROS (*p* < 0.05).

### 3.4. Cecal Bacterial

#### 3.4.1. Cecal Bacterial Community Richness, Diversity, and Composition

Sparse curves based on OTU indicated that most diversity and novel phylotypes were captured (Figure S2A). Meanwhile, the PCoA plot displayed the analysis and demonstrated improved segregation of data between the two treatment groups (Figure S2B), further suggesting that the grouping is reasonable. The differences in the α-diversity index (Figure S2C) between the two treatments showed that SY significantly improved (*p* = 0.011) Shannon indices, with a tendency to increase chao and ace indices (*p* = 0.083, *p* = 0.078).

Clustering at 97% similarity grouped non-repeat sequences into 2340 OTUs; of these, 631 and 265 unique OTUs were found in the SY and SS groups of rations, respectively (Figure 1A). Phylum-level analysis showed that *Firmicutes*, *Bacteroidetes*, and *Verrucomicrobiota* were the dominant species in the cecum, which accounted for more than 84% of all species (Figure 1B). Meanwhile, 19 families had relative abundances more significant than 1%. Among the predominant families were *Lachnospiraceae*, *Lactobacillaceae*, *Prevotellaceae*, and *Oscillospiraceae* (Figure 1B).

#### 3.4.2. Significantly Different Cecal Bacteria Between the SY and SS Groups

Figure 2 summarizes microbial community shifts across the phylum to genus level in the SY versus the SS groups. The relative abundance of *Bacteroidota* was reduced in the SY versus SS group, but the abundance of *Proteobacteria*, *Fusobacteriota* and *Cyanobacteria* was higher in the SY group (Figure 2A). LEfSe analysis revealed genus-level differences in cecal bacteria (Figure 2B). It was shown that SY reduced the relative abundance of the *Lachnospiraceae_AC2044_group*, *Lachnospiraceae_FCS020_group*, *Lachnospiraceae_UCG-003*, *Lachnospiraceae_XPB1014_group*, *Marvinbryantia*, *Rikenellaceae_RC9_gut_group*, *unclassified_f_Lachnospiraceae*, *unclassified_c__Clostridia*, *norank_f_norank_o_Clostridia_UCG-014* and 38 other microorganisms. However, SY improved the relative abundance of *Lactobacillus*, *unclassified_f_Succinivibrionaceae*, *Succinivibrionaceae_UCG-002*, *Agathobacter* and 27 other microorganisms.

### 3.5. Cecal Metabolomics

The overlapping total ion chromatogram of the QC sample in positive (A) and negative (B) ion modes (Figure S3) confirmed the stability and reproducibility of the data obtained herein.

A total of 193 DAMs were detected in the SY vs. SS groups (Figure 3A), including 88 upregulated and 105 downregulated metabolites. The 193 DAMs were subjected to metabolic enrichment and pathway analysis for biochemical pathway mapping (KEGG, http://www.genome.jp/kegg/, accessed on 21 October 2024). After analysis by scipy.stats (Python package 1.2.0: https://docs.scipy.org/doc/scipy/, accessed on 21 October 2024), a total of 130 pathways were enriched, including 48 that reached statistical significance, and the top 20 pathways are presented in Figure 3C, including the Sphingolipid metabolism and Sphingolipid signaling pathway (*p* = 0.016, *p* < 0.001). Key metabolites within this pathway are S1P and sphingosine, which were upregulated in SY1 (Figure 3B).

### 3.6. Cecal Transcriptome

DEGs analysis was performed using DESeq2 (v1.1.56.1; http://bioconductor.org/packages/stats/bioc/DESeq2/, accessed on 21 October 2024) to identify DEGs between comparative groups. DEGs with *p* < 0.05 and fold change (FC) > 1 were defined as upregulated, while those with *p* < 0.05 and FC < 1 were downregulated. This analysis yielded 408 significant DEGs, comprising 209 up- and 199 downregulated genes (Figure 4A). KEGG enrichment analysis of these DEGs was performed using KOBAS (v3.0) with Fisher’s exact test. Pathways with false *p*< 0.05 were considered significantly enriched. Among the 249 enriched pathways, 29 showed markedly significant enrichment (*p* < 0.05). As visualized in Figure 4C, the top 20 significantly enriched pathways are presented. Notably, the PI3K-Akt signaling pathway was highly enriched (*p* = 0.002). Key DEGs within this pathway are displayed in Figure 4B, including upregulated genes (*PIK3AP1*, *GNGT2*).

### 3.7. Spearman Correlation Analysis

#### 3.7.1. Cecal Bacteria Abundance and Growth Performance, Nutrient Digestibility, Serum Biochemical Parameters, Meat Quality, Cecal Fermentation Variables, and Antioxidant and Immune Indicators for Duodenum and Cecum

According to the results, *Lactobacillus*, *unclassified_f_Succinivibrionaceae*, *Succinivibrionaceae_UCG-002* and *Agathobacter* improved in SY and were positively related to ADG, nutrient digestibility, serum biochemical parameters, and cecal fermentation variables (Figure 5A); Se content in longissimus dorsi muscle, WHC, and shear force (Figure 5B); and antioxidant activity. By contrast, they were negatively associated with iNOS, the concentration of MDA and NO, and immune indicators (Figure 5C,D). However, *Lachnospiraceae_AC2044_group*, *Lachnospiraceae_FCS020_group*, *Lachnospiraceae_UCG-003*, *Lachnospiraceae_XPB1014_group*, *Rikenellaceae_RC9_gut_group* and *unclassified_f_Lachnospiraceae* were reduced in SY and exerted an inverse relation to the above indicators.

#### 3.7.2. Cecal Differential Metabolites and Growth Performance, Nutrient Digestibility, Serum Biochemical Parameters, Cecal Fermentation Variables, and Antioxidant and Immune Indicators in Duodenum and Cecum

Furthermore, metabolites including 3a,7a,12a-Trihydroxy-5b-cholestan-26-al, homogentisic acid (HGA), phlorizine, LysoPC(18:1(11Z)/0:0), dethiobiotin, spermine, sphingosine and S1P that were upregulated in the SY group were positively correlated with ADG, nutrient digestibility and cecal fermentation variables (Figure 5E), Se content in the longissimus dorsi muscle and WHC (Figure 5F), and antioxidant activity, while being negatively correlated with iNOS and the concentration of MDA, NO and immune indicators (Figure 5G,H).

#### 3.7.3. Cecal Differential Metabolites and Discrepant Bacteria

As shown in Figure 5G, *Agathobacter* and *unclassified_f_Succinivibrionaceae* were positively related to phlorizine and LysoPC(18:1(11Z)/0:0). Meanwhile, *Agathobacter* and *unclassified_f_Succinivibrionaceae* were positively related to S1P and spermine, respectively. *Lactobacillus* was positively related to HGA. *Succinivibrionaceae_UCG-002* was associated with 3a,7a,12a-Trihydroxy-5b-cholestan-26-al, spermine, and dethiobiotin. *Lachnospiraceae_AC2044_group* and *unclassified_f_Lachnospiraceae* were negatively connected to 3a,7a,12a-Trihydroxy-5b-cholestan-26-al, spermine and sphingosine. A negative relation was identified between *Lachnospiraceae_FCS020_group* and sphingosine. *Lachnospiraceae_UCG-003* and *Lachnospiraceae_XPB1014_group* were negatively related to spermine.

### 3.8. Combined Transcriptome and Metabolome Analysis

Pathway enrichment analysis revealed 67 and 23 pathways significantly enriched with DAMs and DEGs, respectively, in the SY and SS groups. Among these, six pathways demonstrated co-enrichment for both DAMs and DEGs (Figure S4), exemplified by key signaling pathways such as the MAPK pathway (Figure 6); notably, this included the Ras-activating gene *RASGRF1* (Log2FC = 2.320, *p* = 0.012) and the signaling metabolite diacylglycerol (DAG, log2FC = 0.042, *p* = 0.000), both of which were upregulated.

### 3.9. The S1P Content and the Gene Expression of ASAH1, SPHK2, S1PR2, and Key Genes in PI3K-Akt Signaling Pathway in Cecum Mucosa

As shown in Table 6, SY increased the content of S1P and upregulated the mRNA expression of *ASAH1*, *SPHK2*, *S1PR2*, *GNGT2*, *PIK3AP1*, *PIK3CA*, and *Akt1* in the cecum (*p* < 0.001).

## 4. Discussion

Muscle nutritional value, such as the levels of crude protein and crude fat, is a key indicator of meat quality. Se content in muscle works as a key marker of meat nutritional value. Additionally, the critical physical features for judging meat quality are color (L*, a*, and b*), drip loss, and shear force. In general, lower L and b values but higher a* values indicate higher meat quality [26]. Changes in b* may be associated with the process of lipid oxidation [27]. Organic Se has been found to suppress post-oxidative reactions in muscle, thereby enhancing meat’s nutritional quality, taste, storage stability, as well as its color and moisture retention capacity [13]. In our study, compared with SS, SY supplementation significantly increased the CP content, while decreasing EE content in the longissimus dorsi muscle, contributing to improved nutritional value. It also increased Se deposition while reducing b* values, which may be attributed to the suppression of lipid peroxidation. Furthermore, SY supplementation enhanced water-holding capacity and tenderness, as evidenced by reduced drip loss and decreased shear force. Overall, these findings show that SY strengthens the overall quality of donkey meat. Notably, Se deposition in muscle was increased by more than two-fold in Se-supplemented donkeys, suggesting that Se-enriched donkey meat is an essential dietary source of Se for humans. As meat quality is closely associated with antioxidant capacity, we further explored the effects of SY on antioxidant and immune parameters in donkeys.

Redox imbalance occurs when the body’s antioxidant defenses fail to counteract elevated ROS [28]. The greater activity of SOD, CAT, and GPx, and the lower content of MDA, displayed better antioxidant properties in animals [29]. Increased cytokines drive the production of iNOS and NO during the inflammatory process, thereby regulating inflammatory responses [30]. It has been found that the rise in the growth of Agus calves was related to the body’s ability to prevent oxidative stress [31]. Correspondingly, it was concluded that SY improved the CAT, T-SOD, GPx, and T-AOC, and decreased the level of MDA, NO, IL-1β, IL-2, and IL-6, as well as ROS, indicating that SY boosted antioxidant capacity. Elevated CHO and NEFA promote lipid peroxidation *via* mechanisms such as iron-mediated free radical generation and increased oxidative susceptibility of low-density lipoprotein particles [32]. In our study, SY reduced the content of CHO, NEFA, LDL-C, and MDA (the product of lipid peroxidation), which indicated that SY inhibited the lipid peroxidation process and improved the antioxidant capacity, thereby underlying the better meat quality and growth performance.

Prior studies addressing the role of Se inclusion on growth performance have concentrated on cattle, sheep, pigs, and broiler chickens [33,34,35,36]. A previous study by our research group investigated that SY addition to lactating donkeys can promote their lactation performance, antioxidant status, and immune responses [12]. The findings reported here indicate that SY improved the ADG of meat donkeys, which was similar to research on beef cows [37]. Nutrient digestion plays a crucial role in ADG. Li et al. observed that the SY in the ration could notably enhance the digestibility of CP, NDF, and ADF in Shanbei White Cashmere Wether goats [38]. This was explained by the fact that SY improves nutrient digestion by promoting rumen fermentation and regulating rumen flora in Sika Deer [39]. In this work, SY enhanced the digestibility of CP, EE, and NDF, which was consistent with the above studies and further illustrates that SY improves growth by boosting nutrient digestion. BCP concentration indicates protein conversion efficiency and microbiota composition, with NH3-N providing the main nitrogen for BCP synthesis. VFA levels and profiles reflect cecal digestion and metabolism. In this study, SY improved the level of BCP and NH_3_-N in the cecum, suggesting that SY promoted cecal fermentation. These results further explained that SY enhanced the growth of meat donkeys, which could be ascribed to its facilitation of nutrient digestion by promoting cecal fermentation.

The interaction between gut bacteria and the host confers a range of benefits by optimizing nutrient assimilation, preserving intestinal barrier integrity, fostering immune tolerance to antigens, and orchestrating host immunity [40]. A stable gut microbiota is crucial in nutrient digestion and rumen fermentation [41]. Previous evidence indicated that the *Rikenellaceae_RC9_gut_group* notably showed a correlation with CP digestibility, ADG, and serum CAT activity [42]. The present research indicated that SY reduced the *Rikenellaceae_RC9_gut_group*, which further explained its growth-promoting properties.

VFAs reduce inflammatory markers by regulating metabolism in colonic epithelial cells [43]. Among the common SCFAs, butyrate is particularly evident in maintaining gut barrier and immunomodulatory functions [44]. Intestinal butyrate suppresses neutrophil-derived pro-inflammatory cytokine production and dampens inflammation [45]. It was found that propionate and butyrate, given as early as possible after the injury and within the defined therapeutic window, reduced the inflammatory and pro-fibrotic response [46]. Notably, butyrate-producing gut bacteria may exert beneficial effects by suppressing inflammation and regulating both innate and adaptive immunity [47]. *Agathobacter and Lactobacillus* are two bacteria that produce butyrate [48]. Specifically, the genus Lactobacillus significantly alters to regulate the host metabolism and immune system, particularly in preserving intestinal barrier function integrity and exhibiting anti-inflammatory activity by producing tryptophan metabolites and VFAs [49]. *Lactobacillus* was implicated in fiber degradation, modulating immunity, and is key to food digestion and absorption [50]. In this work, the analysis revealed that *Lactobacillus* was positively related to the digestibility of CP and EE. In this study, SY significantly improved *Agathobacter* and *Lactobacillus.* The abundance of beneficial bacteria was positively related to BW, Se and CP content in the longissimus dorsi muscle, butyrate, and CAT and T-AOC, while it was negatively related to EE content, L* and b* of donkey meat, NO, iNOS, IL-1β, and ROS. Furthermore. *Agathobacter and Lactobacillus* were positively connected to S1P, which was upregulated in SY and enriched in sphingolipid metabolism and the sphingolipid signaling pathway. Furthermore, sphingomyelinase degrades sphingomyelin to ceramide on the cell membrane, which is subsequently converted to sphingosine by ceramidase, and then phosphorylated to S1P [51]. ASAH and SPHK are key enzymes coordinating S1P production: ASAH provides sphingosine for SPHK-mediated phosphorylation to S1P, enabling dual regulation of sphingolipid metabolism and extracellular signaling. S1P improves endothelial barrier function and inhibits inflammatory factors and oxidative stress [52]. Moreover, S1P regulated angiogenesis and endothelial barrier integrity [53]. S1P reduced ROS, TNF-α, IL-1β, and IL-6, while elevating IL-10 [54]. S1P primarily signals through its receptors, including S1PR2, which antagonizes apoptosis and cell proliferation and modulates actin remodeling [55]. In the current work, sphingosine and S1P, which were upregulated in the SY group, were positively linked to antioxidant activity, while negatively related to iNOS and the concentrations of MDA, NO, and immune indicators. Furthermore, after targeted validation, SY improved the content of S1P and mRNA expression of *ASAH1*, *SPHK2*, and S1PR2 in the cecum, which provided a conceptual framework for subsequent in vitro validation experiments. In summary, the present results suggest that SY was associated with improved meat quality and growth performance, together with higher S1P levels and greater abundance of butyrate-producing bacteria (*Agathobacter* and *Lactobacillus*). Increased butyrate levels improved cecal barrier integrity and antioxidant function, supporting nutrient utilization, Se deposition, and systemic growth efficiency.

It delineated a critical and conserved role for the PI3K-Akt signaling pathway in mediating cellular protection against oxidative stress and inflammation. Evidence from in vivo models demonstrates that interventions like puerarin treatment enhance the activity of GPx and SOD, while lowering ROS and MDA levels, effects demonstrably dependent on the PI3K-Akt signaling pathway, as their abrogation by specific PI3K-Akt inhibitors confirms [56]. Se protected cardiomyocytes from cell cycle arrest caused by oxidative stress, an effect probably caused by activation of the redox status-associated PI3K-Akt pathway [57]. Accumulating findings have indicated that PI3K-Akt functions as a distal signaling cascade of S1P [58]. In TNF-α-treated HUVECs, the ApoM-S1P complex exerted protective effects against cellular damage and inflammatory response, as evidenced by reduced expression of pyroptosis-related proteins and inflammatory cytokines, along with enhanced PI3K and Akt phosphorylation. These protective effects were reversed by JTE-013, a selective S1PR2 antagonist, indicating that ApoM-S1P signals through S1PR2 to stimulate the PI3K-Akt pathway, thereby mitigating pyroptosis and inflammation [59]. In this work, transcriptomic analysis of cecal tissue revealed significant enrichment of the PI3K-Akt signaling pathway, accompanied by upregulation of the genes *PIK3AP1* and *GNGT2*. Furthermore, qRT-PCR analysis validated that organic Se significantly enhanced the expression of *PIK3AP1*, *GNGT2*, *PIK3CA*, and *Akt1*. These findings further substantiate that organic Se promotes its antioxidant functions in donkeys by activating this pathway.

Additionally, our integrated analysis of the cecal metabolome and transcriptome demonstrated that the MAPK signaling pathway was significantly enriched in both datasets. S1PR2 is primarily associated with Gq and triggers phospholipase C activation, inducing activation of MAPK kinases [60]. This suggests that Se may potentially mediate the entry of S1P into the MAPK signaling pathway, thereby enhancing the antioxidant capacity in donkeys. This finding provides a novel avenue for future research.

Integrating the above analyses, Figure 7 illustrates the proposed hypothetical model linking the observed changes in our study. Dietary SY entering the cecum increases ASAH1 expression, thereby elevating Sph levels. This rise in Sph subsequently upregulated *SPHK2* expression, leading to increased S1P production. S1P then signaled through S1PR2 to activate both the PI3K-Akt and MAPK signaling pathways, ultimately enhancing antioxidant capacity. Concurrently, Sph and/or S1P may be associated with greater abundance of beneficial bacteria and lower abundance of harmful bacteria. This microbial shift resulted in elevated butyrate levels, which also contributed to increased antioxidant capacity.

Several limitations should be acknowledged. Only eight donkeys per group (16 in total) were used owing to limited availability of suitable animals; although individual intake and growth data were recorded, the small sample size limits statistical power and generalizability, and future studies should increase animal numbers and consider breed, sex, and age. In addition, Spearman correlation was used only to link the measured indicators to the differential serum and liver metabolites, and the direct effect of any specific metabolite was not tested. The temporal sequence and causal relationship between microbial and metabolite changes therefore cannot be established from the present data, and these results should be considered exploratory and hypothesis-generating. Furthermore, no yeast-without-Se control was included. Because the yeast carrier contains β-glucans, mannan-oligosaccharides, nucleotides, and yeast proteins that can alter hindgut microbiota (notably *Lactobacillus*), fermentation end-products, and mucosal immune signaling, the observed microbial and fermentation responses cannot be unequivocally attributed to organic Se rather than the carrier, which may overestimate the effect of Se per se; future studies should therefore include a yeast-without-Se or dose-matched yeast matrix control. It should also be noted that both diets contained 0.3 mg/kg of Se and that no basal diet without Se supplementation was included, so this study compared Se sources at an equivalent supplementation level rather than the effect of Se supplementation itself. Finally, the differential metabolite identification and correlation analyses were performed without multiple testing correction, increasing the risk of false positives; these results should therefore be interpreted as exploratory and validated in future work with adjusted significance thresholds.

## 5. Conclusions

In conclusion, our findings collectively demonstrate that dietary organic Se enhances donkey meat quality, including increased Se and CP content, water-holding capacity, and meat color; it also decreases EE content and shear force, as well as systemic antioxidant capacity, and promotes colonization of beneficial cecal microbiota in donkeys. The present findings are consistent with a model in which SY supplementation is associated with higher antioxidant capacity in donkeys, potentially involving increased S1P levels and S1PR2-related PI3K-Akt signaling. Se exhibits the potential to increase Se deposition and improve the quality of donkey meat, thereby supplying an innovative approach for the development of Se-enriched donkey meat products.

## Figures and Tables

**Figure 1:**
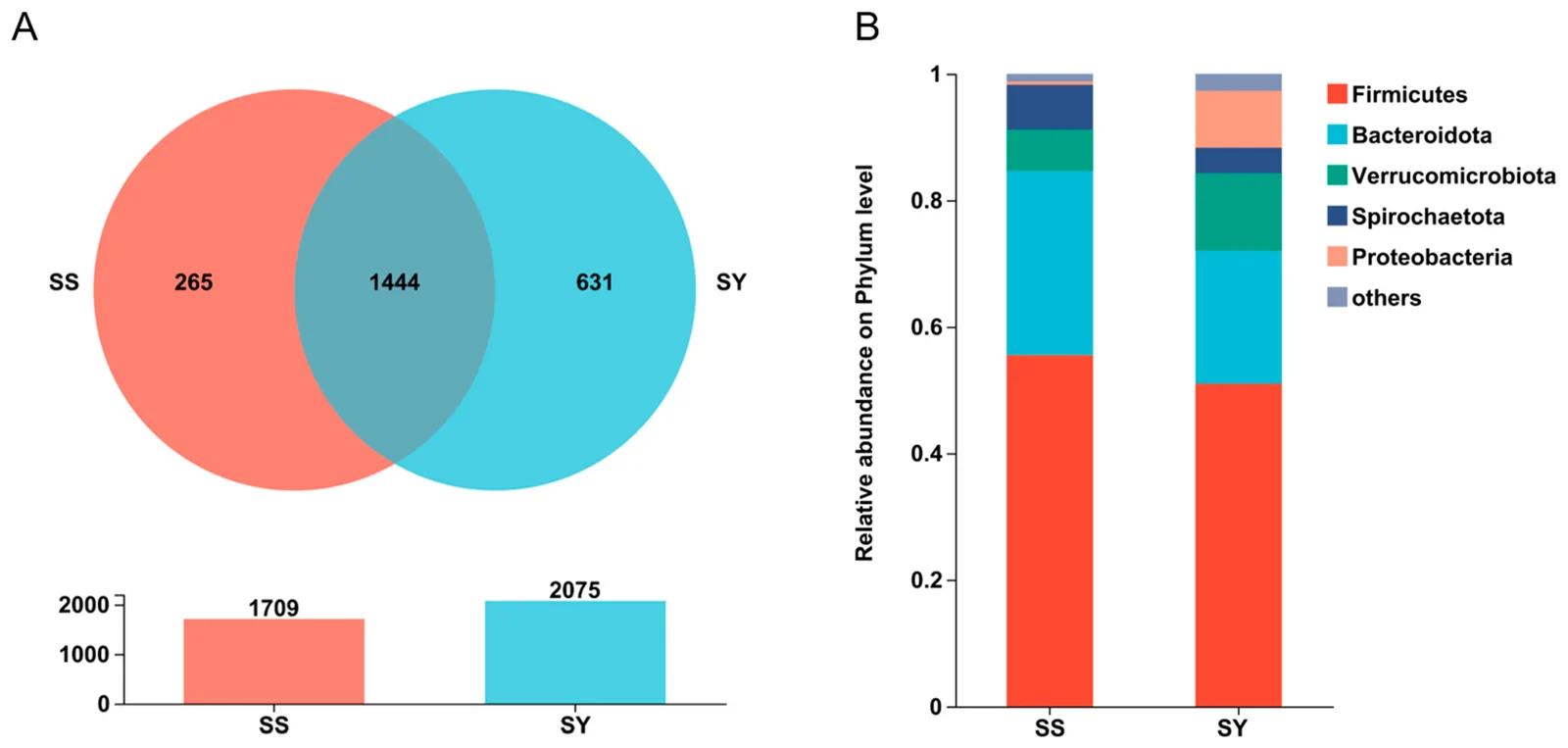
Microbiota compositional profiles of SY and SS in the cecal contents of meat donkeys. (**A**) Venn diagram of the OTUs in the rectal microbiota. (**B**) The relative abundances of rectal bacteria are at the phylum level. SY, yeast selenite group; SS, sodium selenite group.

**Figure 2:**
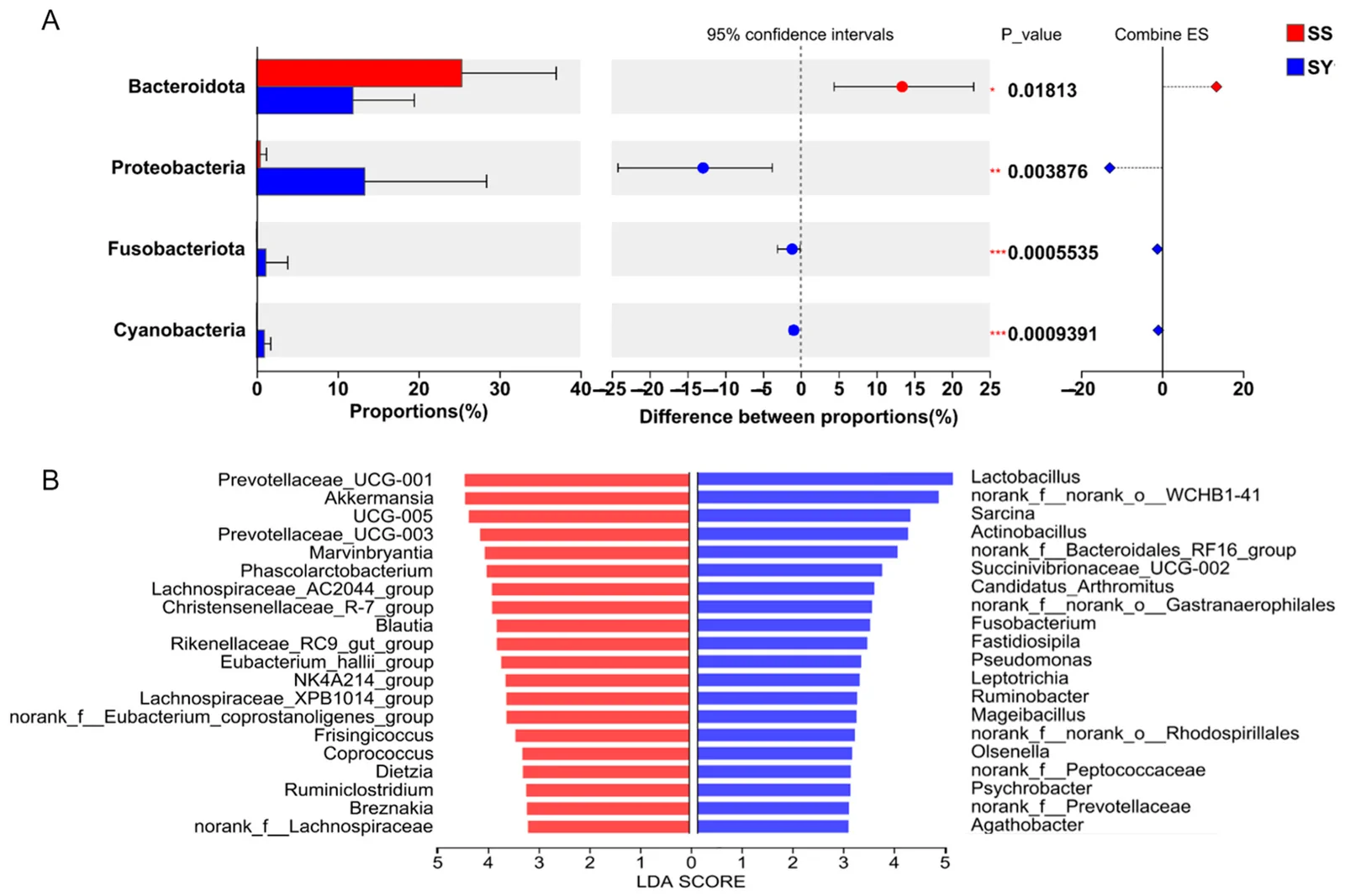
Cecal differential bacteria in meat donkeys. Differential bacterial compositions at the (**A**) phylum and (**B**) genus levels using LefSE analysis of cecal microbiota among 2 dietary treatments. Linear discriminant analysis (LDA) value-distributed histogram; a score ≥ 2.9 is significant. The red curves represent donkeys in the SS (sodium selenite) group, and the blue curves represent donkeys in the SY (yeast selenite) group.

**Figure 3:**
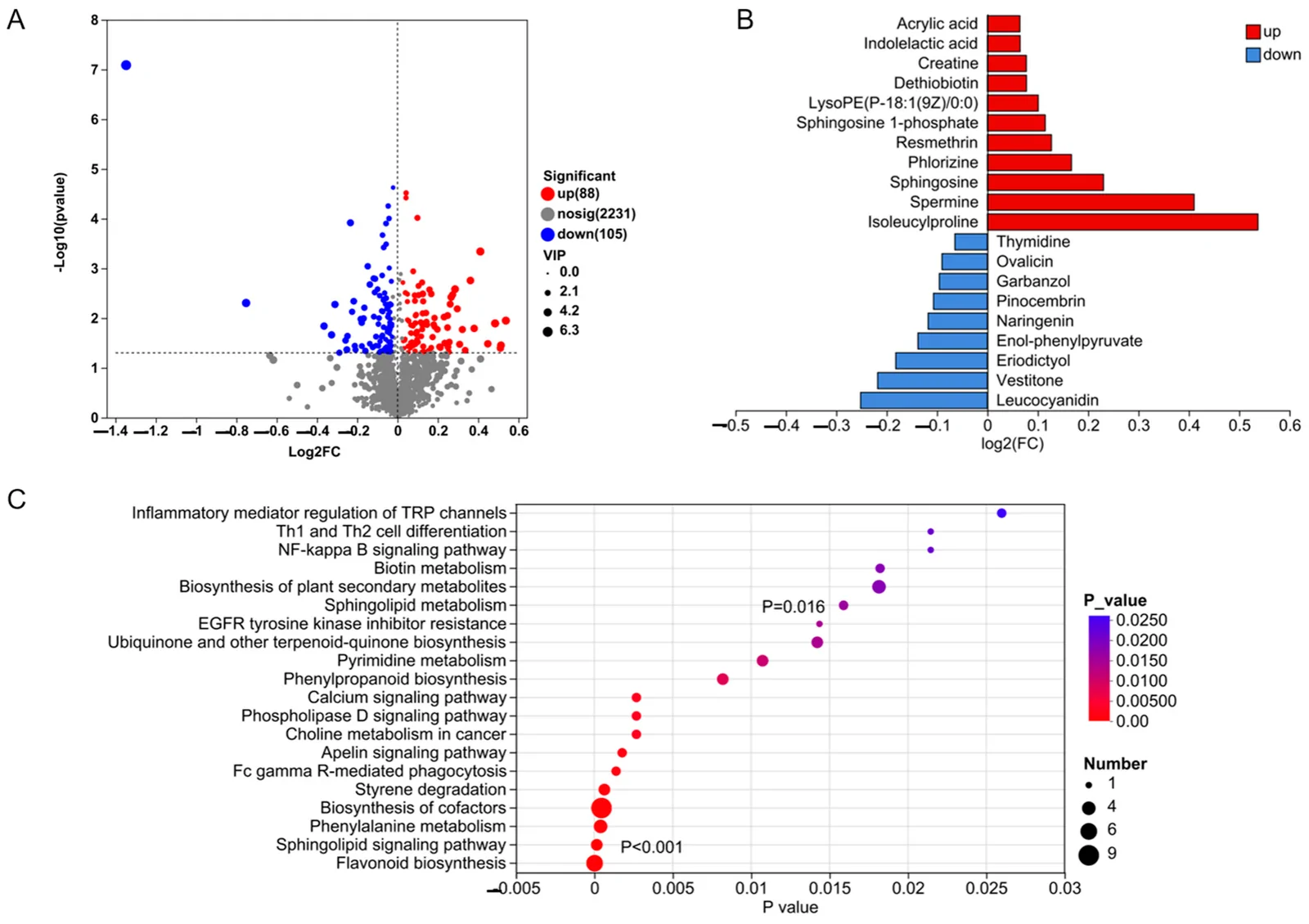
(**A**) Differential metabolites in SY_vs._SS: the red curves represent upregulated metabolites; blue represents downregulated metabolites; and grey represents no change. (**B**) Top 20 significantly differential metabolites for SY_vs._SS. (**C**) Top 20 significantly enriched pathways for SY_vs._SS.

**Figure 4:**
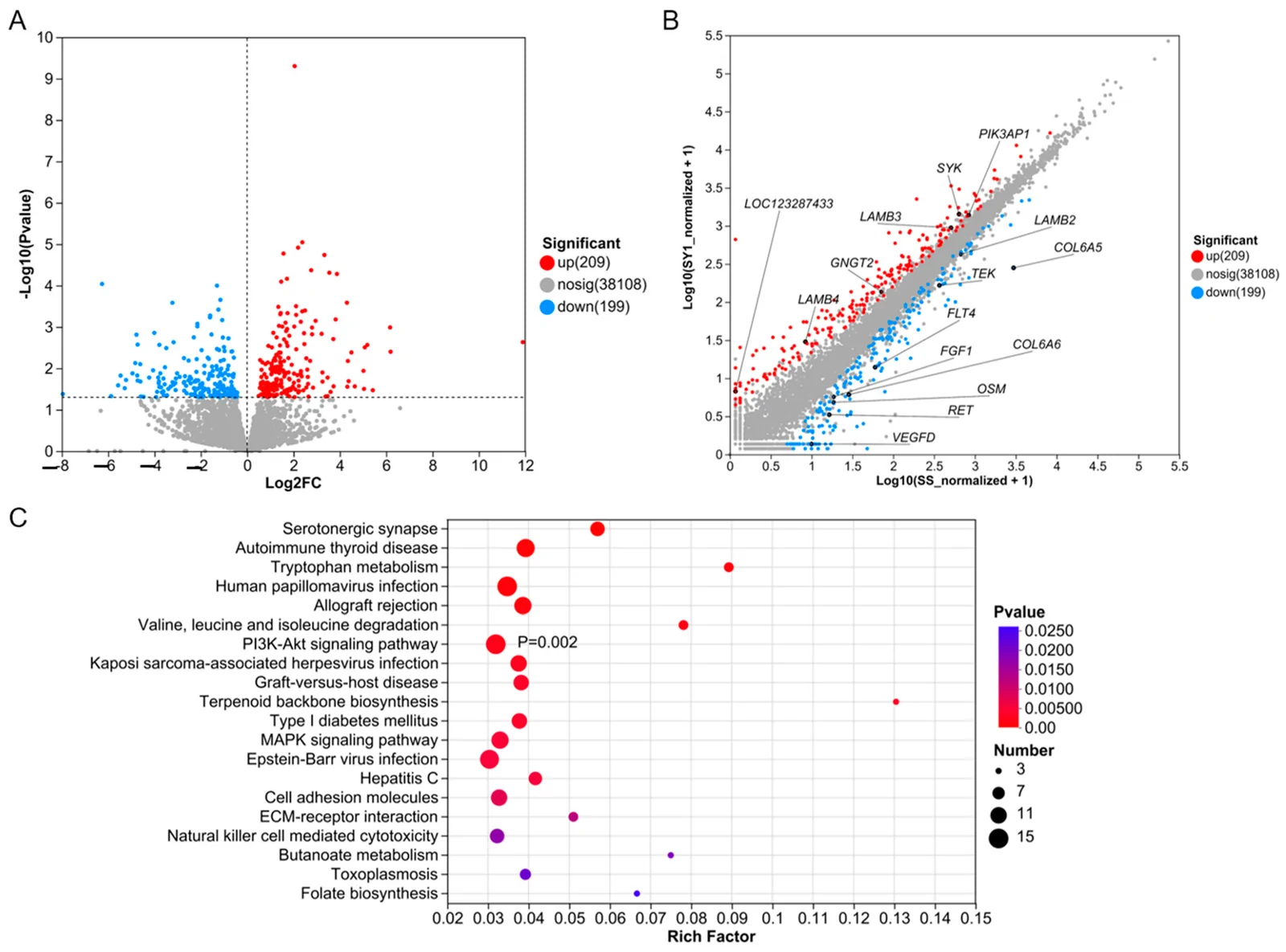
(**A**) Differential genes in SY_vs._SS are represented by the red curves, which indicate upregulation; the blue curves represent downregulation; and the grey curves represent no change. (**B**) Key genes in the PI3K-Akt signaling pathway. (**C**) Top 20 significantly enriched pathways in SY vs. SS.

**Figure 5:**
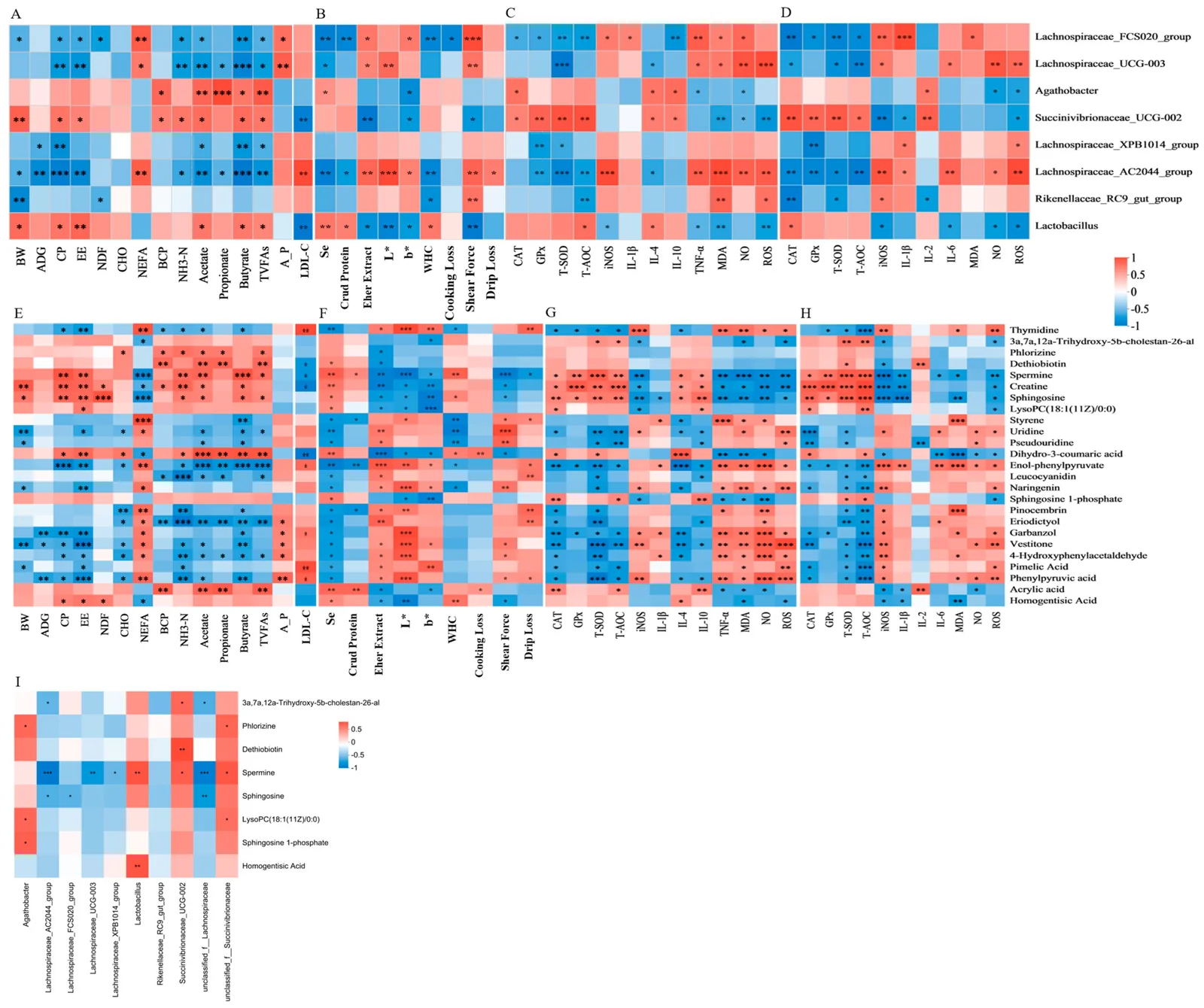
Correlation analysis between cecal bacteria and (**A**) growth performance, nutrient digestibility, serum biochemical parameters, and cecal fermentation variables; (**B**) antioxidant and immune indicators in duodenum; (**C**) meat quality indicators; (**D**) antioxidant and immune indicators in cecum; cecal metabolites and (**E**) growth performance, nutrient digestibility, serum biochemical parameters and cecal fermentation variables; (**F**) meat quality indicators; (**G**) antioxidant and immune indicators in duodenum; (**H**) antioxidant and immune indicators in cecum; and (**I**) cecal bacteria. Red color, positive correlation; blue color, negative correlation; * *p* < 0.05; ** *p* < 0.01; *** *p* < 0.001.

**Figure 6:**
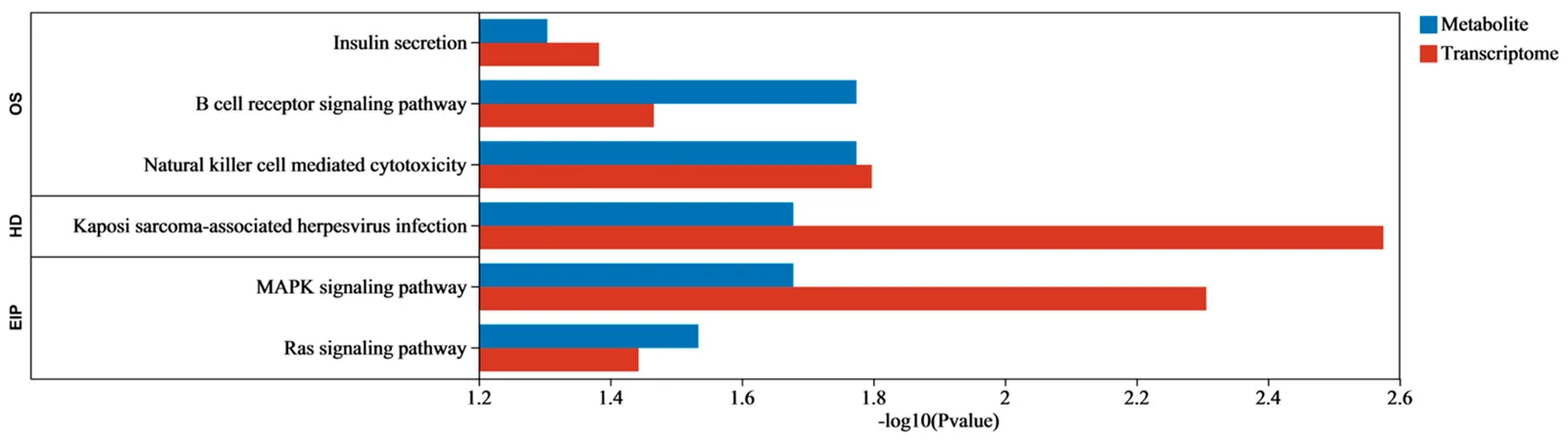
Subset of KEGG pathways co-enriched with DEGs and DAMs.

**Figure 7:**
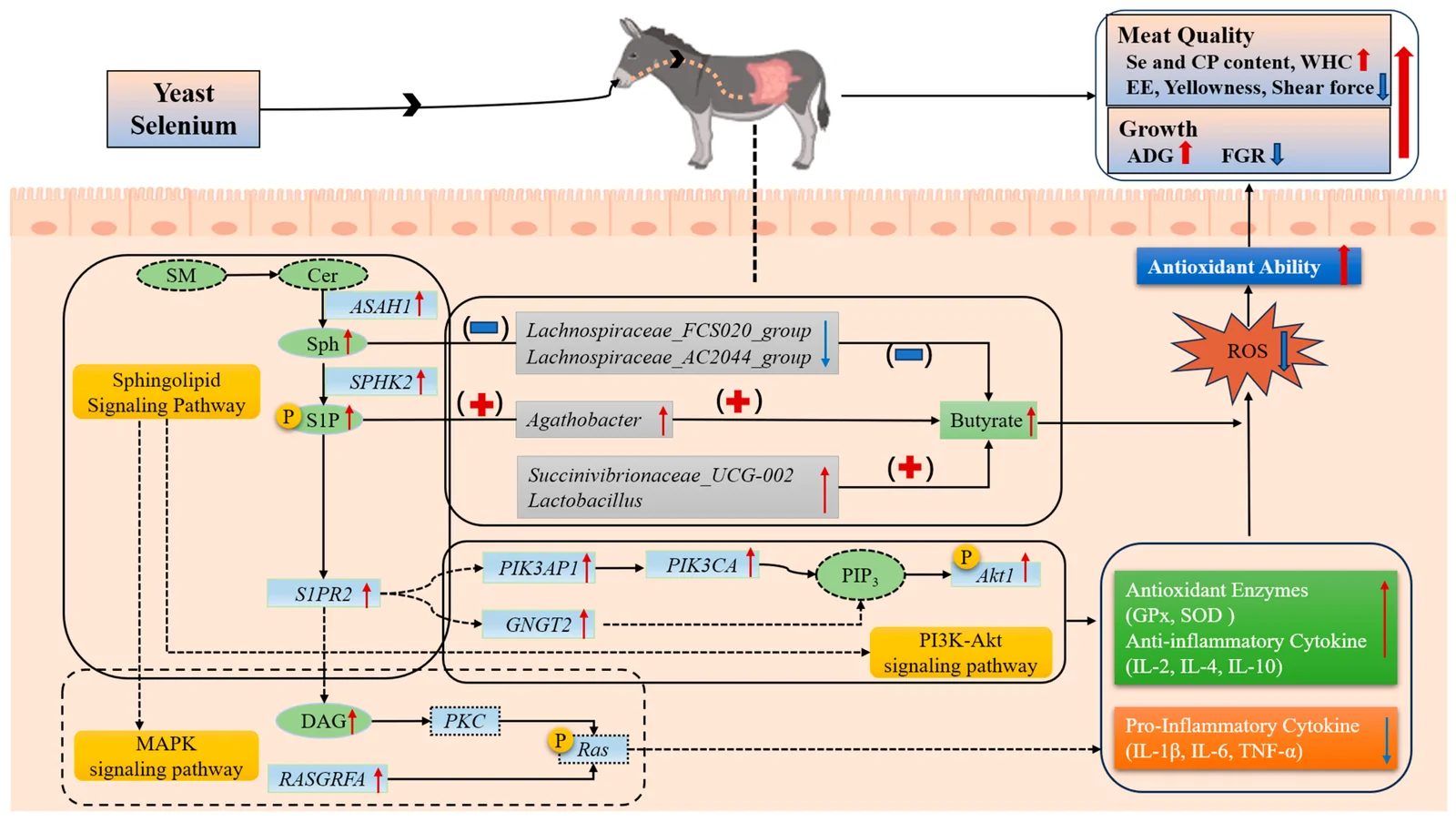
A hypothetical model summarizing the proposed associations among the observed changes in this study. (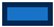) represents a negative relation, (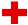) represents a positive relation, (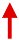) represents an increase, (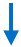) represents a decrease. (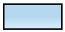) represents genes, (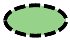) represents metabolites, and (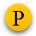) represents phosphorylation.

**Table 1:** Basal diet composition and nutrient level (dry-matter basis, %).

Item	E ^1^	M	L
Ingredient			
Millet straw	36.60	31.10	24.88
Alfalfa	15.00	10.50	8.40
Corn silage	8.40	8.40	6.72
Corn	21.88	33.10	40.20
Wheat midding	0	0	2.58
Soybean meal	8.20	7.80	4.02
Corn germ meal	1.80	1.20	3.66
DDGS	2.00	1.80	2.82
Bran	3.60	0	0
Soybean oil	0.40	0.50	0.84
Puffed full-fat soybeans	0	3.10	3.00
NaCl	0.40	0.40	0.40
Limestone	0.30	0.37	0.44
CaHPO_4_	0.88	1.11	1.32
Premix ^2^	0.24	0.24	0.27
Baking soda	0.30	0.38	0.45
Total	100	100	100
Composition/%			
DE ^3^, MJ/Kg	13.10	13.72	14.30
Crude protein	13.84	13.79	12.78
Ether extract	3.51	4.28	4.83
Neutral detergent fiber	48.23	41.01	35.98
Acid detergent fiber	27.82	23.61	19.70
Calcium	1.08	1.03	0.99
Phosphorus	0.45	0.48	0.54

^1^ E = early fattening; M = middle fattening; L = late fattening. ^2^ Each kg of premix provided the following: Fe 10 g, Cu 2 g, Zn 15 g, Mn 10 g, I 120 mg, Co 60 mg, VA 1 500,000 IU, VD 3 600,000 IU, VE 6000 IU, VK 4460 mg, VB_1_ 80 mg, VB_2_ 1900 mg, VB_6_ 200 mg, nicotinic acid 4500 mg, D-pantothenic acid 3700 mg, VB_12_ 12 mg, biotin 56 mg, and folic acid 510 mg. ^3^ Digestible energy was a calculated value.

**Table 2:** Effects of selenium sources on the growth performance of meat donkeys.

Item		BW, kg	DMI, kg/d	ADG, g/d	FGR
Se	SY ^1^	153.60 ± 12.93 ^A^	4.66 ± 0.72	691.59 ± 46.91 ^A^	7.58 ± 2.88
	SS ^2^	148.99 ± 14.75 ^B^	4.35 ± 0.70	593.71 ± 45.37 ^B^	7.93 ± 1.62
FST ^3^	E	151.29 ± 14.11 ^C^	4.51 ± 0.70 ^C^	642.65 ± 49.11 ^A^	7.75 ± 2.20
	M	178.71 ± 11.80 ^B^	4.85 ± 0.24 ^B^	622.83 ± 35.35 ^A^	8.24 ± 0.79
	L	199.39 ± 12.39 ^A^	5.19 ± 0.46 ^A^	586.66 ± 46.42 ^B^	9.47 ± 0.63
*p*-value	Se	<0.001	0.539	<0.001	0.146
	FST	<0.001	<0.001	0.020	0.634
	Se × FST	0.747	0.955	<0.001	0.978

^1^ SY, selenite yeast group; ^2^ SS, sodium selenite group. ^3^ FST = fattening stage, E = early fattening, M = middle fattening, L = late fattening. ^ABC^ In the same column, values with different capital letters indicate significant differences between the two groups (*p* < 0.05). Data are presented as mean ± SD (n = 8 per group). *p*-values for Se, FST, and Se × FST were obtained from a linear mixed model.

**Table 3:** Effects of selenium sources on the Se content, physicochemical properties, and proximate composition of donkey meat (fresh weight basis).

Item	SY ^1^	SS ^2^	*p* Value
Se content, mg/kg	0.24 ± 0.06 ^A^	0.09 ± 0.03 ^B^	<0.001
Proximate composition, %			
Moisture	74.66 ± 1.01 ^A^	73.43 ± 1.25 ^B^	<0.001
CP	22.97 ± 0.39 ^A^	21.31 ± 0.24 ^B^	0.011
EE	2.23 ± 0.08 ^B^	2.72 ± 0.13 ^A^	<0.001
Physicochemical properties, %			
pH45min	7.06 ± 0.25	7.01 ± 0.14	0.644
pH24h	5.41 ± 0.07	5.33 ± 0.11	0.187
L*	26.66 ± 2.00 ^B^	28.71 ± 0.44 ^A^	<0.001
a*	13.47 ± 1.07	13.14 ± 0.61	0.441
b*	6.18 ± 0.68 ^B^	7.52 ± 0.66 ^A^	<0.001
Water-holding capacity	7.24 ± 0.67 ^A^	6.20 ± 0.40 ^B^	<0.001
Cooking loss	36.41 ± 0.82 ^A^	35.19 ± 0.77 ^B^	0.019
Shear force	0.86 ± 0.36 ^B^	1.21 ± 0.16 ^A^	<0.001
Drip loss	12.44 ± 0.51 ^B^	13.33 ± 0.51 ^A^	0.012

^1^ SY, selenite yeast group; ^2^ SS, sodium selenite group. ^AB^ In the same column, values with different capital letters indicate significant differences between the two groups (*p* < 0.05). Data are presented as mean ± SD (n = 8 per group). *p*-values were obtained from independent-samples *t*-tests.

**Table 4:** Effects of selenium sources on nutrient digestibility, serum biochemical indices, and cecal fermentation function of meat donkeys.

Item	SY ^1^	SS ^2^	*p* Value
Serum biochemical parameters, mmol/L			
GLU	5.40 ± 1.14	5.43 ± 1.45	0.966
CHO	2.19 ± 0.23 ^B^	1.92 ± 0.24 ^A^	0.038
TG	0.09 ± 0.02	0.08 ± 0.01	0.633
NEFA	0.44 ± 0.06 ^B^	0.55 ± 0.05 ^A^	<0.001
HDL-C	2.55 ± 0.59	2.60 ± 0.55	0.757
LDL-C	0.68 ± 0.27 ^B^	1.29 ± 0.51 ^A^	0.002
Nutrient digestibility, %			
CP	84.17 ± 1.78 ^A^	80.02 ± 3.18 ^B^	0.001
EE	78.59 ± 1.52 ^A^	73.77 ± 3.40 ^B^	<0.001
NDF	51.18 ± 8.24 ^A^	43.81 ± 5.71 ^B^	0.020
ADF	43.8 ± 6.36	40.92 ± 8.59	0.450
Cecal fermentation function			
pH	6.64 ± 0.22	6.75 ± 0.24	0.328
BCP, mg/100 mL	14.55 ± 3.62 ^A^	9.28 ± 3.30 ^B^	0.006
NH_3_-N, mg/100 mL	9.69 ± 2.32 ^A^	6.96 ± 1.74 ^B^	0.013
Volatile fatty acids, mmol/L			
Acetate	42.27 ± 4.21 ^A^	29.10 ± 3.44 ^B^	<0.001
Propionate	16.47 ± 3.03 ^A^	11.26 ± 1.21 ^B^	0.001
Isobutyrate	0.28 ± 0.05	0.34 ± 0.12	0.265
Butyrate	4.57 ± 0.60 ^A^	2.87 ± 0.40 ^B^	<0.001
Isovalerate	0.30 ± 0.04 ^A^	0.22 ± 0.13 ^B^	0.008
Valerate	0.49 ± 0.01 ^A^	0.41 ± 0.03 ^B^	0.000
TVFA	63.53 ± 5.92 ^A^	44.31 ± 4.13 ^B^	<0.001
A/P	2.46 ± 0.16 ^B^	2.60 ± 0.15 ^A^	0.033

^1^ SY, selenite yeast group; ^2^ SS, sodium selenite group. ^AB^ In the same column, values with different capital letters indicate significant differences between the two groups (*p* < 0.05). Data are presented as mean ± SD (n = 8 per group). *p*-values were obtained from independent-samples *t*-tests.

**Table 5:** Effects of selenium sources on antioxidant and immune indicators of the duodenum and cecum in meat donkeys.

Item	SY ^1^	SS ^2^	*p* Value
Duodenum			
Enzyme activity, U/mgprot.			
CAT	11.61 ± 4.17 ^A^	7.24 ± 1.84 ^B^	0.001
GPx	89.38 ± 1.63 ^A^	74.52 ± 4.63 ^B^	0.03
T-SOD	298.55 ± 5.40 ^A^	162.40 ± 4.43 ^B^	<0.001
T-AOC	18.18 ± 3.47 ^A^	8.02 ± 2.13 ^B^	<0.001
iNOS	18.22 ± 7.16 ^B^	25.60 ± 7.04 ^A^	0.005
Cytokine content, pg/mgprot.			
IL-1β	1.85 ± 0.19 ^B^	1.97 ± 0.15 ^A^	0.032
IL-2	13.47 ± 0.59	12.68 ± 1.18	0.234
IL-4	1.16 ± 0.14 ^A^	0.98 ± 0.09 ^B^	0.012
IL-6	7.17 ± 0.14	7.31 ± 0.13	0.546
IL-10	1.79 ± 0.14 ^A^	1.55 ± 0.07 ^B^	0.001
TNF-α	3.62 ± 0.28 ^B^	4.63 ± 0.12 ^A^	<0.001
MDA concentration, nmol/mgprot.	0.58 ± 0.05 ^B^	0.94 ± 0.17 ^A^	<0.001
NO concentration, umol/mgprot.	4.57 ± 0.81 ^B^	6.70 ± 0.58 ^A^	<0.001
ROS level, IU/mgprot.	82.59 ± 2.02 ^B^	88.29 ± 2.50 ^A^	0.001
Cecum			
Enzyme activity, U/mgprot.			
CAT	10.11 ± 1.89 ^A^	6.29 ± 0.78 ^B^	<0.001
GPx	89.38 ± 1.63 ^A^	74.52 ± 4.63 ^B^	0.042
T-SOD	351.23 ± 4.12 ^A^	273.81 ± 4.10 ^B^	0.001
T-AOC	16.04 ± 3.86 ^A^	9.09 ± 1.27 ^B^	<0.001
iNOS	3.54 ± 1.44 ^B^	7.61 ± 1.02 ^A^	<0.001
Cytokine content, pg/mgprot.			
IL-1β	4.58 ± 0.27 ^B^	4.89 ± 0.50 ^A^	0.007
IL-2	79.74 ± 3.52 ^A^	70.19 ± 2.68 ^B^	0.012
IL-4	0.85 ± 0.03	0.82 ± 0.04	0.074
IL-6	52.52 ± 6.99 ^B^	58.43 ± 4.39 ^A^	0.006
IL-10	1.40 ± 0.15 ^B^	1.95 ± 0.15 ^A^	<0.001
TNF-α	8.38 ± 0.45 ^A^	7.49 ± 0.31 ^B^	0
MDA concentration, nmol/mgprot.	0.27 ± 0.04 ^B^	0.41 ± 0.13 ^A^	0.002
NO concentration, umol/mgprot.	5.74 ± 0.61 ^B^	6.28 ± 0.46 ^A^	0.004
ROS level, IU/mgprot.	277.61 ± 4.15 ^B^	313.26 ± 4.83 ^A^	<0.001

^1^ SY, selenite yeast group; ^2^ SS, sodium selenite group. ^AB^ In the same column, values with different capital letters indicate significant differences between the two groups (*p* < 0.05). Data are presented as mean ± SD (n = 8 per group). *p*-values were obtained from independent-samples *t*-tests.

**Table 6:** Effects of SY on the content of S1P and expression of S1P-relevant genes key genes within the PI3K-Akt signaling pathway in the cecum mucosa of donkeys.

Item	SY ^1^	SS ^2^	*p* Value	SEM
S1P content, nmol/gprot.	805.16 ^A^	603.75 ^B^	<0.001	22.542
Gene expression				
*ASAH1*	2.26 ^A,3^	1.00 ^B^	<0.001	0.112
*SPHK2*	1.42 ^A^	1.00 ^B^	<0.001	0.021
*S1PR2*	2.36 ^A^	1.00 ^B^	<0.001	0.002
*GNGT2*	1.23 ^A^	1.00 ^B^	<0.001	0.030
*PIK3AP1*	1.44 ^A^	1.00 ^B^	<0.001	0.008
*PIK3CA*	1.07 ^A^	1.00 ^B^	<0.001	0.011
*Akt1*	1.34 ^A^	1.00 ^B^	<0.001	0.050

^1^ SY, selenite yeast group; ^2^ SS, sodium selenite group. ^AB^ In the same column, values with different capital letters indicate significant differences between the two groups (*p* < 0.05). ^3^ Gene expression values represent fold change normalized to the SS group.

## Data Availability

The raw sequencing data generated have been stored in the China National GeneBank Database (CNGBdb) under accession numbers CNP0007725 (microbiome), CNP0007726 (metabolome), and CNP0007727 (transcriptome). All relevant information is available in the manuscript or Supplementary Materials.

## Supplementary Materials

The following supporting information can be downloaded at https://www.mdpi.com/article/10.3390/foods15203589/s1, Figure S1: Gels and blots of genes. Abbreviations: ASAH1 = acid ceramidase 1; SPHK2 = sphingosine kinase 2; S1PR2 = sphingosine-1 phosphate receptor 2; GNGT2 = guanine nucleotide-binding protein subunit gamma-T2; PIK3AP1 = phosphoinositide 3-kinase adapter protein 1, PIK3CA = phosphatidylinositol-4,5-bisphosphate 3-kinase catalytic subunit-alpha; Akt1 = RAC serine/threonine-protein kinase 1; GAPDH = glyceraldehyde-3-phosphate dehydrogenase; Figure S2: Microbiota compositional profiles of SY and SS in the cecal contents of meat donkeys. (A) The OTU rarefaction curves; (B) PCoA (using the Bray–Curtis similarity metric) of bacterial OUT; (C) α-diversity index. AB Means that 2 groups that do not have a common marked letter differ significantly (*p* < 0.05). SY, yeast selenite group; SS, sodium selenite group; Figure S3: (A) Cecal content ion chromatogram in Pos. (B) Cecal content ion chromatogram in Neg. (C) OPLS-DA score plots of SY_vs._SS in Pos. (D) OPLS-DA score plots of ME vs. HE in Neg. (E) Permutation test of OPLS-DA model for group SY_vs._SS in Pos. (F) Permutation test of OPLS-DA model for group SY_vs._SS in Neg; Figure S4: Venn diagrams of the number of pathways annotated and enriched by DAMs and DEGs; Table S1: The Primers for qRT-PCR.
