# Supplementary material for: Effects of Selenium Sources on Meat Quality and Antioxidative Capacity in Donkeys: An Integrated Study Combining Cecal Microbiota Profiling and Multi-Omics Analysis
Source: Foods. 2026 Oct 9;15(20):3589. doi: 10.3390/foods15203589 (PMC13703270; doi:10.3390/foods15203589)
Supplement: Supplementary file 1 [file foods-15-03589-s001.zip › foods-4560271-supplementary.pdf]

Effects of Selenium Sources on Meat Quality and Antioxidative Capacity in Donkeys: An Integrated Study Combining Cecal Microbiota Profiling and Multi-Omics Analysis

Li Li, Yanli Zhao *, Xiaoyu Guo, Yongmei Guo, Qingyue Zhang, Fanzhu Meng, Fang Hui and Sumei Yan *

Key Laboratory of Animal Nutrition and Feed Science, Universities of Inner Mongolia Autonomous Region, Hohhot 010018, China; lily972021@163.com (L.L.); gxy_2594@163.com (X.G.); ymguo2015@163.com (Y.G.); alicezqy@126.com (Q.Z.); fzmeng2023@163.com (F.M.); cf18447051697@163.com (F.H.)

* Correspondence: ylzhao2010@163.com (Y.Z.); yansmimau@163.com (S.Y.)

Analytical methods for physicochemical characteristics of the longissimus dorsi muscle

1. Meat color: Within 30 min post-slaughter, two muscle samples (5 cm^2 area, 1 cm thickness) were collected and placed in sample holders. The lightness (L), redness (a), and yellowness (b*) values were measured using a colorimeter.

2.pH value (pH_{45min} and pH_{24h}): At 45 min and 24 h post-slaughter, a portable pH meter was inserted into three different positions of the muscle at a depth of 0.5–1 cm. The average value of the three measurements was calculated.

3. Pressing loss (water loss rate): Determined between 30 and 45 min post-slaughter. Meat samples (1 cm thickness) were obtained using a circular sampler (5 cm^2 area) and weighed to record the pre-pressing weight. The sample was then sandwiched between 18 layers of medium-speed qualitative filter paper, with hard plastic plates placed on both sides of the filter paper. A pressure of 35 kg was applied for 5 min. After removing the pressure, the sample was immediately weighed to record the post-pressing weight. Pressing loss (%) was calculated as: (pre-pressing weight – post-pressing weight) / pre-pressing weight × 100%.

4. Drip loss: At 45 min post-slaughter, muscle samples were cut along the direction of muscle fibers into small meat strips (1.5 × 1.5 × 3 cm). After accurate weighing, each strip was hung with a metal wire at one end with the muscle fibers oriented vertically, and placed inside a plastic bottle without contacting the bottle wall. The bottle was stored at 4°C for 24 h. The sample was then removed, surface moisture was blotted dry with filter paper, and the sample was accurately weighed. Drip loss (%) was calculated as: (initial weight – weight after dripping) / initial weight × 100%.

5. Cooking loss: Cooking loss was determined using the meat samples after drip loss measurement. The meat strips were placed in cooking bags and left at room temperature for 15 min, then immersed in 80°C water until the internal core temperature reached 72°C. After maintaining this temperature for 5 min, the samples were removed, cooled to room temperature, and weighed. Cooking loss (%) was calculated as: (initial weight – weight after cooking) / initial weight × 100%.

6. Shear force: Three longissimus dorsi muscle samples placed in self-sealing bags were stored at 4°C for 24 h, then transferred to a –20°C freezer until analysis. Before measurement, the samples were placed in a 71°C water bath for 35 min, then removed and cooled to room temperature, followed by storage at 4°C overnight. Each sample was cut into 5 cm × 1 cm × 1 cm strips and subjected to shear force analysis using a shear force apparatus at a speed of 200 mm/min. Three strips were prepared from each muscle sample, with two cuts per strip, and the average shear force value of six measurements was calculated.

Table S1 The Primers for qRT-PCR

Target	Primer sequences (5′-3′) [1]	GenBank no.	Length (bp)
ASAH1	F: CTGTTTTTGGCGTGACGGTT	XM_014836464.3	175
	R: GCCCACAGAGAATCCTGCTT		
SPHK2	F: GAAACCTCTGACTCCACGCT	XM_070499176.1	118
	R: GTTGCAGCAAGATCACGTCC		
SIPR2	F: GAACAGACAGCCGGATACTC	XM_014857582.3	253
	R: TGAATCCTCTGCCAAGTGAGG		
GNGT2	F: GACCCACTTCTCAAAGGCGT	XM_014833785.2	107
	R: AGGGACAGACGTTGAGGTGA		
PIK3CA	F: GTCAATTGGTGACTGCGTGG	XM_014838359.3	115
	R: TAGCGTGTGGCTGTTGAACT		
PIK3AP1	F: GATGCCGAGGACCCCATTT	XM_044753258.1	157
	R: AGCCTGGTGGTAATAACAGGTG		
Akt1	F: AAGGAGATCATGCAGCACCG R: CTCCATCGTGTCGTCTTGGT	XM_046647544.1	180
	R: CTCCATCGTGTCGTCTTGGT		
β-actin	F: GTGGAGGGACTCATGACCAC	XM_014834961.1	173
	R: AGCTTCCCATTCAGCTCAGG		
GAPDH	F: GATGCCCCAATGTTTGTGA	NM_001163856.1	250
	R: AAGCAGGGATGATGTTCTGG		

Abbreviations: *ASAH1*=acid ceramidase 1; *SPHK2*=sphingosine kinase 2; *S1PR2*=sphingosine-1 phosphate receptor 2; *GNGT2*=guanine nucleotide-binding protein subunit gamma-T2; *PIK3AP1*=phosphoinositide 3-kinase adapter protein 1, *PIK3CA*=phosphatidylinositol-4,5-bisphosphate 3-kinase catalytic subunit-alpha; *Akt1*=RAC serine/threonine-protein kinase 1; *GAPDH*=Glyceraldehyde-3-phosphate dehydrogenase.

[1] F = Forward primer; R = Reverse primer

A

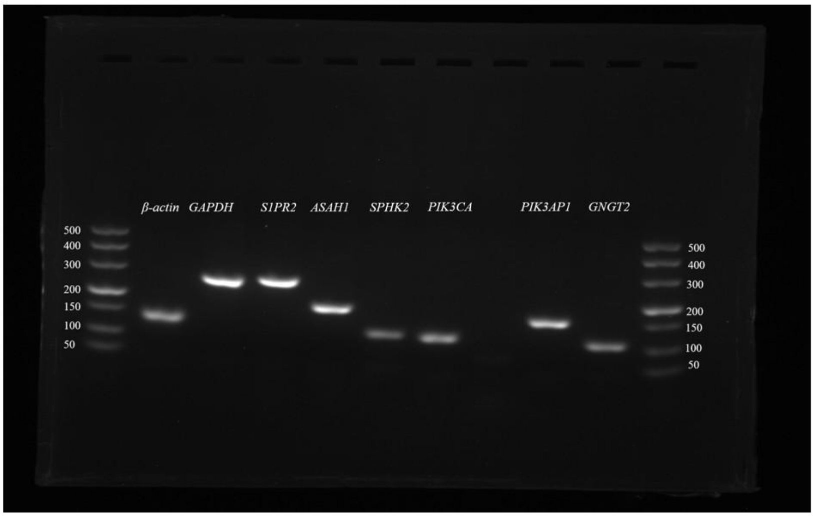

B

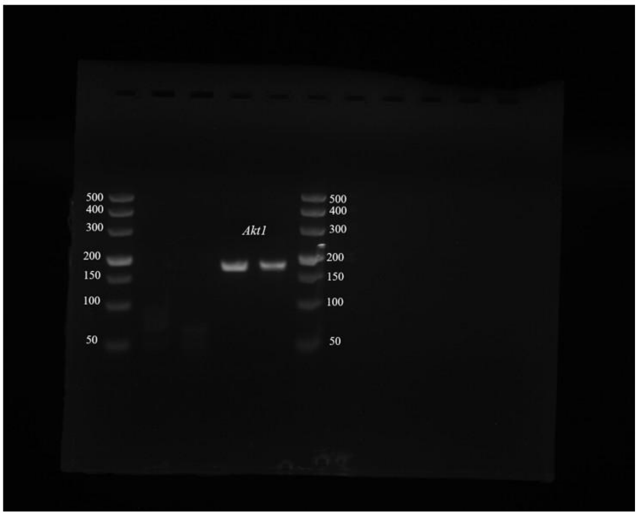

Figure S1. Gels and Blots of genes. Abbreviations: *ASAH1* = acid ceramidase 1; *SPHK2* = sphingosine kinase 2; *S1PR2* = sphingosine-1 phosphate receptor 2; *GNGT2*=guanine nucleotide-binding protein subunit gamma-T2; *PIK3AP1* = phosphoinositide 3-kinase adapter protein 1, *PIK3CA* = phosphatidylinositol-4,5-bisphosphate 3-kinase catalytic subunit-alpha; *Akt1* = RAC serine/threonine-protein kinase 1; *GAPDH* = Glyceraldehyde-3-phosphate dehydrogenase.

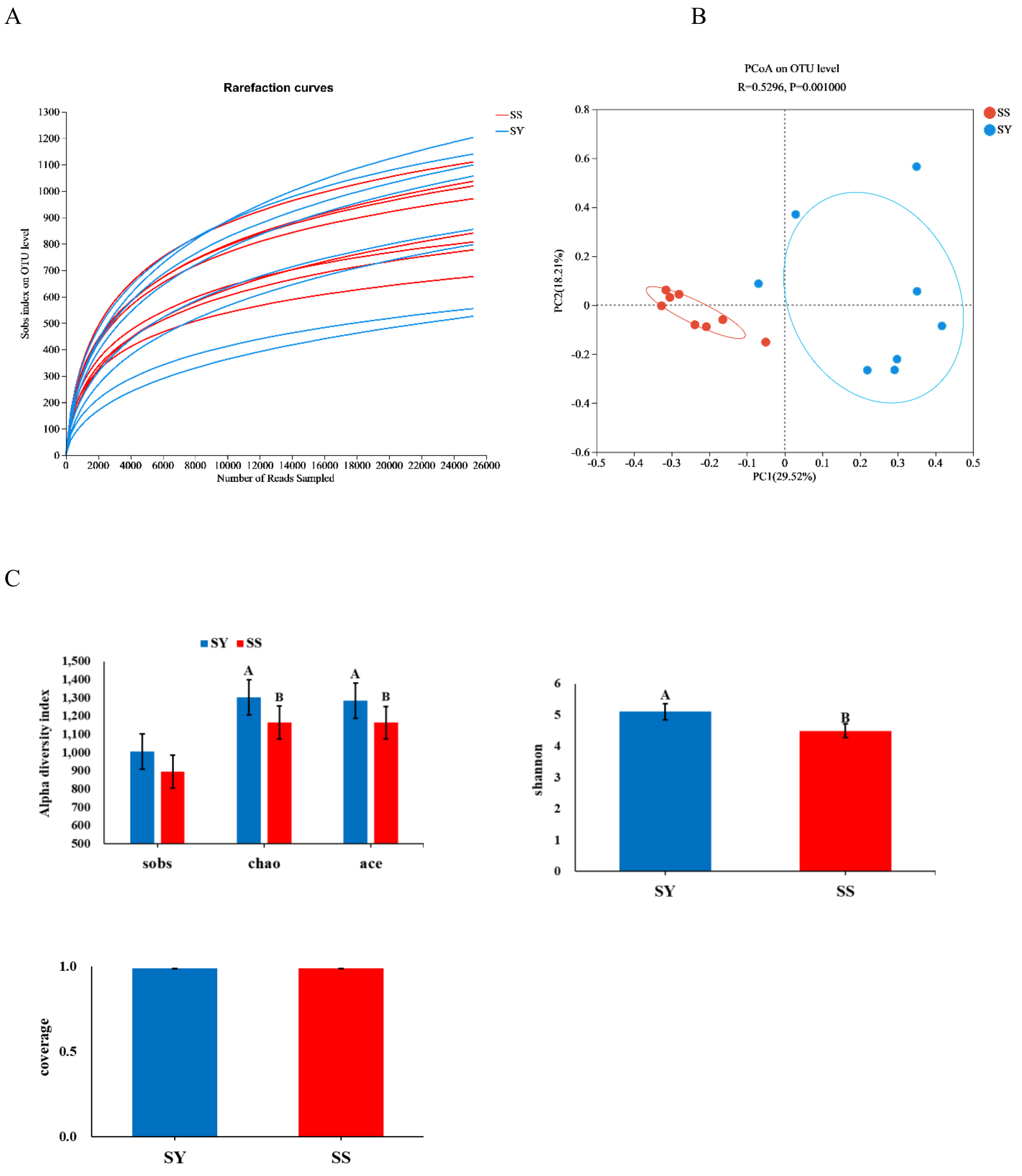

Figure S2 Microbiota compositional profiles of SY and SS in the cecal contents of meat donkeys. (A)The OTU rarefaction curves; (B) PCoA (using the bray Curtis similarity metric) of bacterial OUT; (C) α-diversity index. [AB] Means that 2 groups that do not have a common marked letter differ significantly (P < 0.05). SY, yeast selenite group; SS, sodium selenite group.

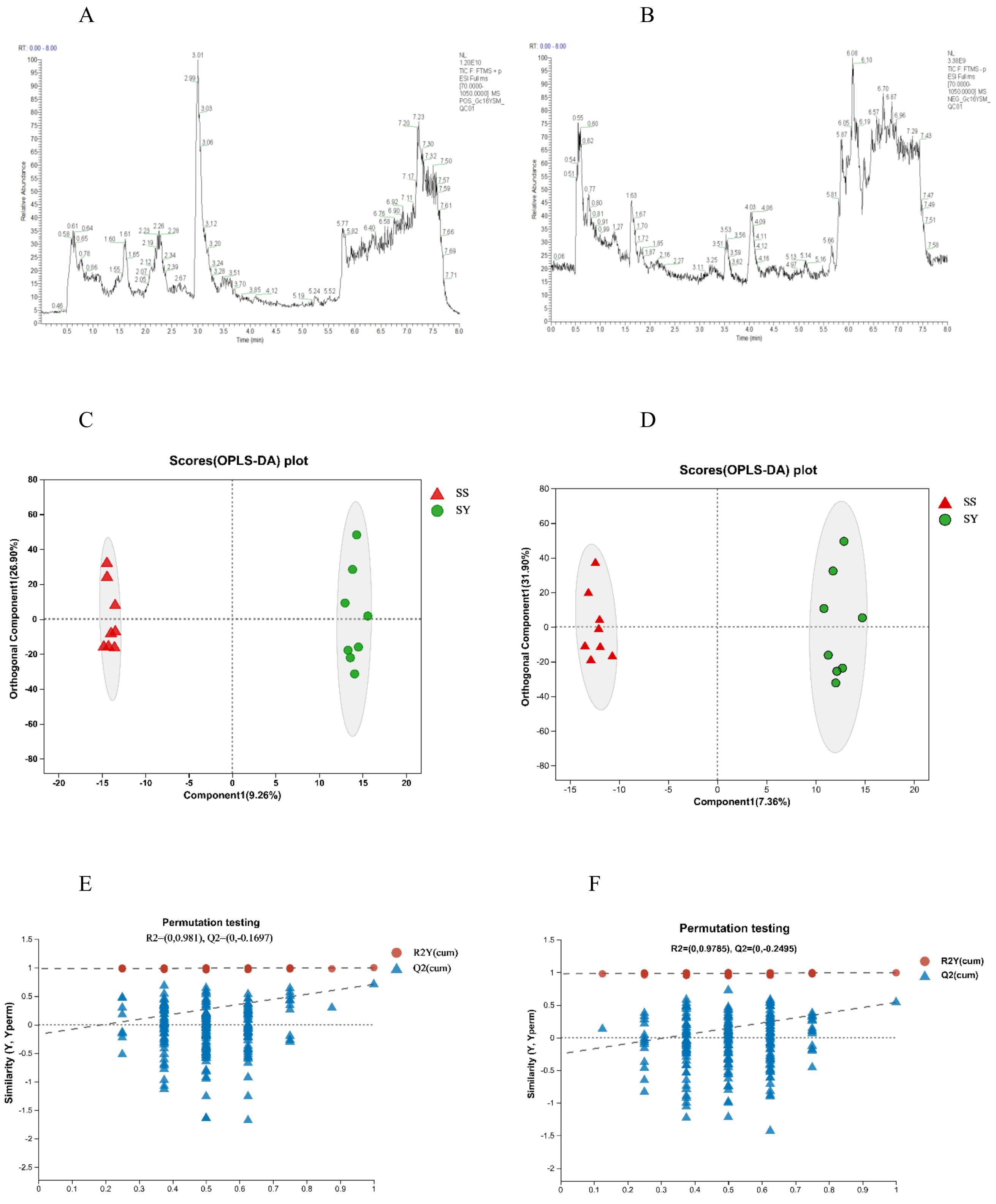

Figure S3 (A) cecal content Ion chromatogram in Pos. (B) cecal content Ion chromatogram in Neg. (C) OPLS-DA score plots of SY_vs._SS in Pos. (D) OPLS-DA score plots of ME vs. HE in Neg. (E) Permutation test of OPLS-DA model for group SY_vs._SS in Pos. (F) Permutation test of OPLS-DA model for group SY_vs._SS in Neg.

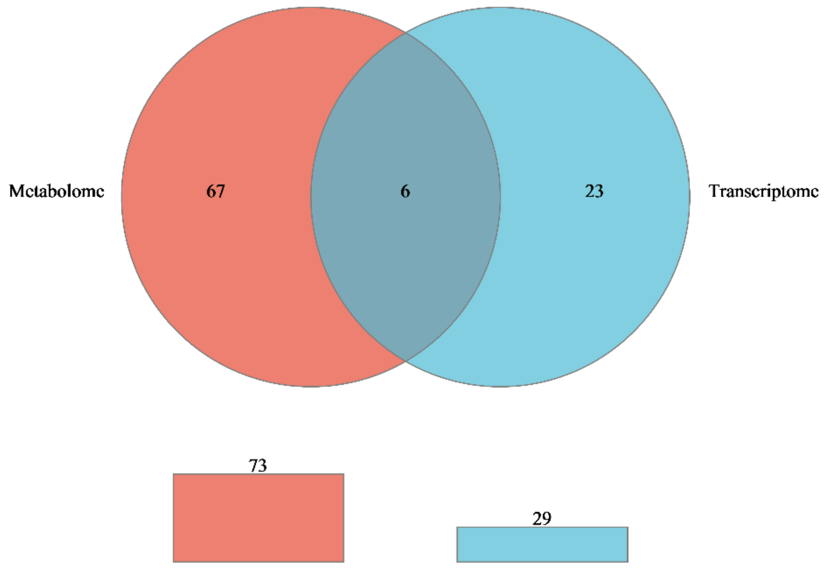

Figure S4 Venn diagrams of the number of pathways annotated and enriched by DAMs and DEGs
